# Online MR guided dose escalated radiotherapy for organ preservation in distal rectal cancer

**DOI:** 10.1016/j.ctro.2022.10.003

**Published:** 2022-10-15

**Authors:** Simon Boeke, Laura Uder, Jakob Ehlers, Sarah Butzer, Sabrina Baumeister, Jessica Boldt, Marcel Nachbar, Monica Lo Russo, David Mönnich, Konstantin Nikolaou, Daniel Zips, Daniela Thorwarth, Cihan Gani

**Affiliations:** a Department of Radiation Oncology, University Hospital and Medical Faculty, Eberhard Karls University Tübingen, Tübingen, Germany; b Section for Biomedical Physics, Department of Radiation Oncology, University Hospital and Medical Faculty, Eberhard Karls University Tübingen, Tübingen, Germany; c Department of Diagnostic and Interventional Radiology, University of Tübingen, Germany; d Department of Radiation Oncology, Berlin Institute of Health, Charité - Universitätsmedizin Berlin, Corporate Member of Freie Universität Berlin, Humboldt-Universität zu Berlin, Berlin, Germany; e German Cancer Consortium (DKTK), Partner Site Tübingen, and German Cancer Research Center (DKFZ), Heidelberg, Germany

**Keywords:** Rectal cancer, MRL, Dose escalation, Adaptive radiotherapy, Organ preservation

## Abstract

**Introduction:**

Non-surgical management of rectal cancer aiming for organ-preservation is an important development to improve rectal cancer treatment. Dose escalated radiotherapy represents one approach to increase clinical complete response (cCR) rates. In the present study we present feasibility and outcome data on rectal cancer patients who were treated with dose escalated radiotherapy using an MR guided online response-adaptive workflow.

**Material and methods:**

A total of five patients were treated with 45 Gy in 25 fractions to the mesorectum and the internal iliac lymph nodes and a simultaneous integrated boost to the primary tumor with 50 Gy in 25 fractions on a conventional linac. In addition, weekly response-adaptive boost fractions with 3 Gy per fraction were scheduled on a 1.5 T MR-Linac. Concomitant chemotherapy with 5-fluorouracil was given as continuous venous infusion during the first and last week of treatment. Response was evaluated approximately-three months after the end of treatment and surgery was omitted in case of a clinical complete response (cCR) or a near cCR. Toxicity was graded by using PRO-CTCAE, Quality of life by the EORTC-QLQ-C30 questionnaire and continence according to the Wexner scale.

**Results:**

Response-adaptive dose escalated radiotherapy was feasible and well tolerated by all patients. Four reached a clinical complete response, one had a local excision confirming pathological complete response (pCR). All PRO-CTCAE grade 3 toxicities resolved within six months after the end of treatment. Quality of life and continence scores during follow-up were comparable to baseline levels.

**Conclusion:**

Dose-escalated online response-adaptive MR-guided radiotherapy appears to be a very promising treatment with the goal of organ preservation in rectal cancer leading to high response rates, excellent organ function and limited side effects. Further prospective evaluation is needed.

## Highlights

• First series of MR-guided response adaptive dose escalated radiotherapy in rectal cancer.
• High patent acceptability with low acute toxicity and promising response rates.

## Introduction

Non-surgical management in case of clinical complete response after radiochemotherapy is now widely considered an acceptable alternative to major surgery [1]. Dose escalated radiotherapy was previously found to increase pathological complete response rates after multimodality treatment [2]. However, due to high inter- and intrafractional positional variability of the rectal tumors and the pelvic organs at risk and the incapability to apply online adaptive treatments, large safety margins to account for this positional variability were necessary in the past using cone beam computed tomography (CB-CT) based treatment devices. This limited the doses that can safely be given [3]. With the recent advent of MR-Linac hybrid devices, it became possible to treat such tumors in a daily adaptive manner and with minimal safety margins [4]. Furthermore, the MR component of the MR-Linac hybrid devices facilitates an excellent soft tissue contrast in the abdomen and pelvis allowing a reproducible and rapid segmentation [5], [6]. Our group has previously developed a novel workflow for MR guided online adaptive dose escalated radiotherapy of rectal primaries with the goal of organ preservation [7], [8]. In the present work we present feasibility and early data on toxicity, quality of life and response in rectal cancer patients treated with dose escalated online response adaptive radiotherapy with the goal of organ preservation.

## Material and methods

In this study we included five patients with distal rectal tumors UICC stage I/IIA who would require abdominopelvic extirpation and permanent colostomy in case of surgery. All patients were treated within a basket trial (NCT04172753) approved by the ethics committee of the medical faculty in Tübingen (659/2017BO1) and written informed consent was given by the patients. All patients received radiotherapy to the primary tumor, the mesorectum and the internal iliac lymph nodes (CTV1) according to standard guidelines for target volume definition of rectal cancer. A safety margin of 7 to 10 mm was used to account for positional uncertainties. CTV1 was treated with 1.8 Gy per fraction to a total of 45 Gy and a simultaneous integrated boost (SIB) to the primary tumor was applied with 2 Gy per fraction to a total of 50 Gy (CTV2). This treatment was scheduled on a conventional non-adaptive linear accelerator with CB-CT image guidance (Versa HD, Elekta, Stockholm, Sweden). Additionally, patients were planned for a weekly response adaptive boost fraction with 3 Gy per fraction on the MR-Linac (Unity, Elekta, Stockholm, Sweden) at least 6 h apart from the previous or the subsequent fraction. In case of early tumor shrinkage and the tumor volume of less than 5 ccm it was at the discretion of the treating physician to conclude the weekly boost fractions and continue just with the fractionated treatment. We have reported the methodology of the online adaptive MR-guided weekly boost fractions previously [7]. Briefly, patients were prepared for the boost fractions with the rectal application of 100 ccm of commercially available ultrasound gel. After this the patient was scanned on the MR-Linac using an anatomical T2w-MR scan (T2w-TSE, FOV: 400 × 400 × 300 mm3, voxel size: 1.5 × 1.5 × 2.0 mm^3^, TE/TR = 278/1535 ms, WFS/BW = 0.293 pix/740.3 Hz, scan time = 1:57 min) which was used for target volume definition and adaptation. GTV to PTV margin of 2 mm cranially and anteriorly were used and 5 mm in all other directions. These margins were based on a previous report of our group and others and the observation that due to bladder filling during treatment target volume shifts were primarily observed in the inferior and posterior direction [7], [9]. A verification scan at the end of plan adaptation was acquired to ensure, that the target did not move during plan optimization. Continuous monitoring of the target coverage was done with orthogonal bFFE images at 5 Hz during beam delivery. Continuous venous infusional 5-FU of 1000 mg / m^2^ body surface area per day over 120 h was given during the first and last week of treatment. We used a previously published patient experience questionnaire in order to evaluate device specific aspects, which was handed to the patients on every boost fraction [10]. For evaluation, every boost fraction was counted as a single datapoint per item. Toxicity, quality of life and incontinence were scored using the patient reported outcome version of the CTCAE questionnaires (PRO-CTCAE), the EORTC-QLQ-C30 questionnaire and the Wexner score. The first assessment of response after radiotherapy took place three months after the end of radiotherapy by rectoscopy and pelvic MRI. Response was graded into a “clinical complete response”, “a near complete response” and a “poor response” based on endoscopy. In order to meet criteria for a CCR no finding other than a scar with telangiectasia was allowed. In case of a residual ulcer patients were considered as near complete responders and in case of macroscopically residual tumors as poor responders. In case of a “near complete response” or a “clinical complete response” patients remained under surveillance and did not undergo immediate surgery. MRI was reserved for the assessment of lymph nodes in the pelvis.

## Results

### Feasibility, toxicity and quality of life

**Fig. 1:**
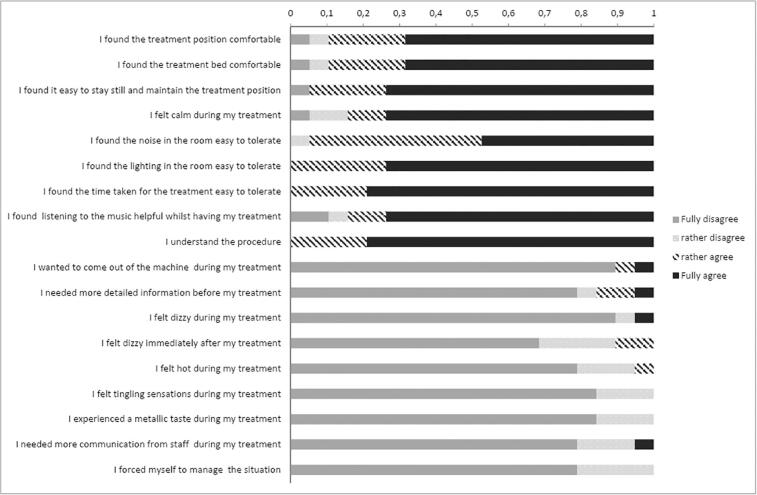
Patient reported outcome for treatment comfort and acceptability.

**Fig. 2:**
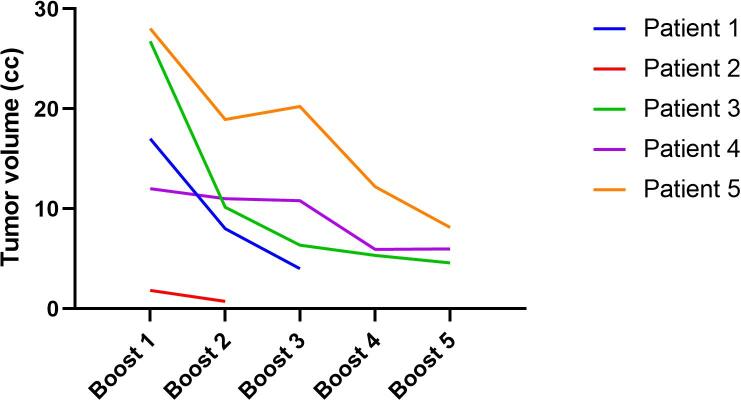
Tumor volumes at the individual boost fractions.

**Fig. 3:**
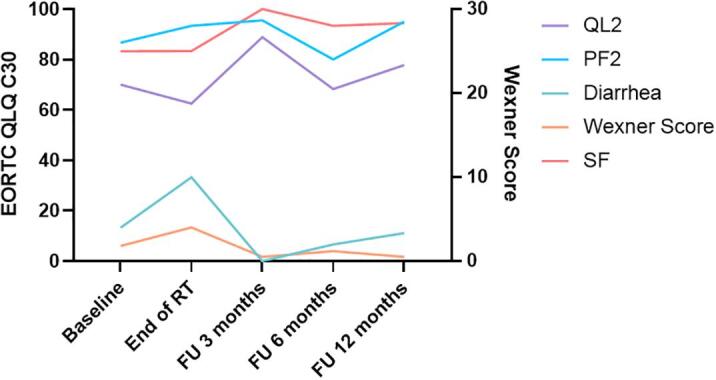
Quality of life and incontinence scores assessed by the EORTC-QLQ-C30 questionnaire and the Wexner score. FU- Follow-up, QL2 - Overall quality of life, PF2-Physical functioning, SF-social functioning.

Among the five reported patients, four had cT3a/b tumors, one primary tumor was staged cT2. The distal end of the primary tumor was in very close proximity to the dentate line in all cases. No patient had clearly suspicious lymph nodes or distant metastases. The mesorectal fascia was affected in two patients. The median (range) volume of the primary tumor was 17 cc (1.9–28 cc). All patients completed radiotherapy to CTV1 and CTV2 as prescribed. Regarding the boost fractions on the MR-Linac, three patients received the maximum number of five boost fractions to a total of 65 Gy. In the remaining two patients one received two and the other three fractions respectively due to considerable tumor shrinkage during treatment resulting in 56 and 59 Gy to the macroscopic tumor. As shown in Fig. 1 patients found “it easy to stay still and maintain the treatment position” despite the rectal filling with ultrasound gel in more than 90 % of all fractions. Tumor volumes at the time of the individual boost fractions are shown in Fig. 2. PRO-CTCAE grade 3 toxicity during treatment was reported by one of the five patients for “diarrhea”, “abdominal pain”, “dysuria”, “fatigue” and “nausea”. PRO-CTCAE grade 3 “urinary frequency” was reported by two patients. However, all grade 3 toxicities resolved at 6-month follow-up. Quality of life assessed by the EORTC QLQ-C30 is shown in Fig. 3. The mean Wexner score improved from 1.8 at baseline to 0.5 at the end of treatment.

### Response

**Fig. 4:**
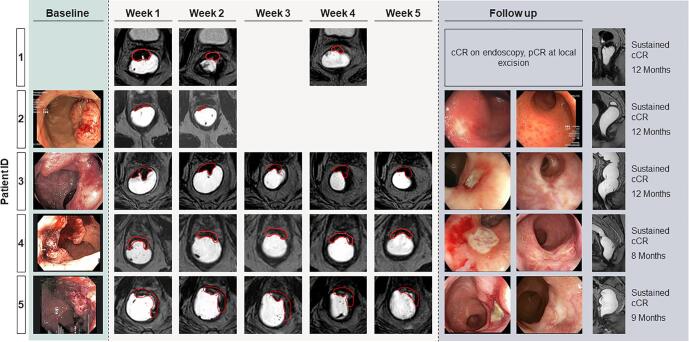
Endoscopic findings (if available) and exemplary MR-images with the delineated GTV for each boost fraction.

At the time of this report none of the patients underwent radical surgery. Patient 1 underwent local excision three months after the end of radiotherapy due to some minor mucosal irregularities. Pathological evaluation of the specimen confirmed a pathological complete response with no viable cells. Three of the four remaining patients achieved a clinical complete response six months after the end of radiotherapy, patient 3 regressed to a clinical complete response at 12 months after having a near complete response at 9 months. Endoscopy and MRI scans at baseline, during treatment and during follow-up are summarized in Fig. 4.

## Discussion

In the present study we report the first cohort of patients who were treated with an online response adaptive dose escalation protocol on an MR-Linac hybrid device. The indication for dose escalation in these patients was to achieve a clinical complete response and avoid a surgical procedure that would require permanent colostomy. With this novel approach for precision radiation oncology, we were able to show an excellent response to treatment in these tumors and a very favorable toxicity profile during radiotherapy and at follow-up. Furthermore, organ function regarding continence scores was preserved. Various different treatment strategies have shown to increase complete response rates in the past [11]). Over the last couple of years multiple trials have established total neoadjuvant therapy as a new standard of care for locally advanced rectal cancer [12], [13] Besides the decrease in distant metastases with the addition of induction or consolidative chemotherapy a considerable increase in complete response rates was also observed in both the RAPIDO and the PRODIGE-23 trial. Therefore, the intensification of the systemic component of treatment is also considered a viable option in order to facilitate organ preservation in rectal cancer. This was impressively confirmed by the OPRA trial in which long-term organ preservation was achieved in approximately 60 % of the patients who received radiochemotherapy followed by 3 cycles of FOLFOX [14]. However, it should be noted that the previously mentioned trials were designed for patients with locally advanced tumors or even high-risk tumors with adverse features such as involvement of the mesorectal fascia or T4 tumors. For patients with early tumors and therefore a lower risk for distant metastases such as in the present report, alternative approaches to increase complete response rates with a more favorable toxicity profile are critically needed in order to avoid the systemic side effects of chemotherapy. For this purpose, dose escalated radiotherapy is the most intuitive treatment as potential side effects are limited to organs at risks in the immediate proximity. This is of utmost importance as previous studies have shown that patients with distal rectal tumors prioritize treatment related side effects very high when it comes to intensified treatment regimens [15]. Yet the effectiveness of dose escalated radiotherapy in rectal cancer was recently questioned by the randomized RECTAL-BOOST trial which failed to show a higher PCR rate after dose escalated radiotherapy. However, the actually applied doses in the experimental arm were lower than planned, which was due to organ at risk constraints and large safety margins to cover day to day anatomical variability [3]. The very favorable toxicity profile observed in the current report is likely to be explained by the small safety margins facilitated by the online adaptive workflow. Furthermore, and based on a previous report by Bonomo et al., we opted for a weekly boost rather than an upfront boost as in the RECTAL-BOOST trial since this approach significantly reduced the dose to organs at risk [4]. Finally, the use of rectal ultrasound gel led to a distancing of the uninvolved rectal mucosa with consecutive lower doses to this area and a pronounced reduction in circumferential dose. Our study does have some limitations. The sample size is small and therefore does not permit any firm conclusions regarding the effectiveness of this treatment. Furthermore, follow-up is still short and a longer observation period is required to assess regrowth rates, long-term organ function and late side effects. This is of particular relevance for patients who would usually not need radiotherapy prior to surgery and might therefore be overtreated if surgery is still required after dose escalated radiotherapy. A randomized controlled trial evaluation a dose escalated radiotherapy protocol as in this trial is currently being prepared.

## Conclusion

In summary, in this first-in-human pilot trial, we were able to show the feasibility of an MR guided weekly adaptive radiotherapy dose escalation regime leading to high complete response rates and very limited side effects.

## Declaration of Competing Interest

The Department of Radiation Oncology Tübingen receives financial and technical support by Elekta, Philips, Siemens, Dr. Sennewald Medizintechnik, Kaiku Health, TheraPanacea, PTW Freiburg and ITV in the context of research cooperations. Travel costs were covered by Elekta for CG, SB.
