# Supplementary material for: Online MR guided dose escalated radiotherapy for organ preservation in distal rectal cancer
Source: Clin Transl Radiat Oncol. 2022 Oct 15;37:153–6. doi: 10.1016/j.ctro.2022.10.003 (PMC9630771; doi:10.1016/j.ctro.2022.10.003)
Supplement: Supplementary data 1 [file mmc1.pdf]

Supplemental table 1

PRO-CTCAE Grade 3 Toxicities

		Patient 1	Patient 2	Patient 3	Patient 4	Patient 5
GI	Diarrhea	-	-	+	-	-
	Abdominal pain	-	-	-	-	+
	Nausea	-	+	-	-	-
GU	Dysuria	-	-	-	-	+
	Increased frequency	-	-	+	-	+
	Fatigue	-	+	-	-	-

No grade III/IV PRO-CTCAE toxicities reported at 6 months follow-up
